# Ising-like Magnetism in Quasi-Two-Dimensional Co(NO_3_)_2_·2H_2_O

**DOI:** 10.3390/ma15207066

**Published:** 2022-10-11

**Authors:** Anna A. Vorobyova, Igor L. Danilovich, Igor V. Morozov, Alexander N. Vasiliev, Olga S. Volkova, Asif Iqbal, Badiur Rahaman, Tanusri Saha-Dasgupta

**Affiliations:** 1 Department of Low Temperature Physics and Superconductivity, M.V. Lomonosov Moscow State University, Moscow 119991, Russia; 2 Quantum Functional Materials Laboratory, National University of Science and Technology “MISiS”, Moscow 119049, Russia; 3 Department of Physics, Aliah University, Kolkata 700156, India; 4 Department of Condensed Matter, S.N. Bose National Centre for Basic Sciences, Kolkata 700106, India

**Keywords:** Ising-like magnetism, low-dimensional magnetic systems, cobalt dinitrate dihydrate

## Abstract

The appearance of electrically neutral water molecules in the structure of cobalt dinitrate dihydrate, Co(NO_3_)_2_⋅2H_2_O, drastically changes its magnetic properties as compared to its waterless counterpart, Co(NO_3_)_2_. The title compound shows Ising-like behavior reflected in its thermodynamic properties. It experiences long-range antiferromagnetic order at *T_N_* = 20.5 K and metamagnetic transition at *µ*_0_*H_C_* = 0.76 T. First-principles calculations produce the values of leading exchange interactions *J1* ~ 10 K and *J2* ~ 0.5 K and single-ion anisotropy *D* ~ 1 K which allows us to consider Co(NO_3_)_2_⋅2H_2_O as a quasi-two-dimensional magnetic system.

## 1. Introduction

Magnets exhibiting field-induced transitions can be divided into highly anisotropic Ising and weakly anisotropic Heisenberg ones. Isotropic materials allow the rotation of the local moment directions perpendicular to the external magnetic field. Phase transitions in anisotropic materials are characterized by reversals of the local moment [1]. The reduction in the dimensionality of the magnetic subsystem manifests itself in different ways in isotropic and anisotropic magnets. The theoretical background of two-dimensional magnetism has been provided by the seminal research of Onsager, who predicted that a monolayer Ising magnet could transit into the long-range ordered state [2]. The essence of the Ising model is the presumption that the magnetic moment of any given atom possesses only one degree of freedom pointing either up or down along a direction dictated by single-ion anisotropy. This is in contrast with the Heisenberg model, which presumes the presence of three degrees of freedom concerning the direction of the magnetic moment. Note that the Mermin–Wagner theorem [3], which postulates the absence of long-range order in isotropic two-dimensional Heisenberg systems, does not apply to the Ising ones. Despite the fundamental importance of Onsager prediction, experimental realizations of ordered two-dimensional magnets were found to be rather rare. In this respect, the discovery of intrinsic ferromagnetism in two-dimensional van der Waals crystals is of the utmost interest [4]. An overview of the progress in the field of two-dimensional magnets is given in Ref. [5].

With this work, we bring attention to the quasi-two-dimensional Ising-like system, Co(NO_3_)_2_⋅2H_2_O, featuring square layers of Co^2+^ ions linked by the nitrate groups. The only studied compound within the *TM*(NO_3_)_2_⋅2H_2_O family, where *TM* is the 3*d* transition metal, is nickel dinitrate dihydrate Ni(NO_3_)_2_⋅2H_2_O [6]. The interlayer coupling in this system is organized by hydrogen bonds. It orders antiferromagnetically at low temperatures [7] and possesses sizable single-ion anisotropy [8]. We present basic thermodynamic properties, i.e., magnetization, *M*, and specific heat, *C_p_*, and first-principles calculations of an exchange interaction parameter, *J_i_*, and single-ion anisotropy, *D*, in layered Co(NO_3_)_2_⋅2H_2_O, which orders antiferromagnetically and indicates metamagnetic behavior. Further, we discuss the properties of Co(NO_3_)_2_⋅2H_2_O in comparison with its waterless counterpart Co(NO_3_)_2_.

## 2. Experiment

Polycrystalline Co(NO_3_)_2_⋅2H_2_O was prepared by the hydrothermal method. Cobalt nitrate dihydrate was preliminarily obtained as a result of partial dehydration of the hexahydrate Co(NO_3_)_2_⋅6H_2_O at a temperature of about 100 °C. To clean as prepared sample of dihydrate from impurities of other hydrates and hydrolysis products, anhydrous HNO_3_ was added to it and the resulting mixture was heated in an autoclave up to 105 °C for 2 h, kept at this temperature for 1 day, and cooled down to 90 ℃ for 5 days. The autoclave was then inverted and held at 90 ℃ for 1 additional day to separate the crystals from the solution. Due to the hygroscopicity of the obtained Co(NO_3_)_2_⋅2H_2_O, further preparation of samples for the physical measurements took place in a dry box in an argon atmosphere with a moisture content of less than 0.1 ppm. According to XRD analysis (Cu K_α_), the sample does not contain impurities, indexing of the XRD powder pattern allows estimating unit cell parameters in a space group *P2*_1_/*n*, with *a* = 6.0170(2), *b* = 8.6331(1), *c* = 5.7353(2) Å, *β* = 92.6625(1)°, *V* = 297.60(5) Å^3^, *Z* = 2 (see Figure S1 of Electronic Supplementary Information), which is in fair agreement with Reference [6].

**Figure 1:**
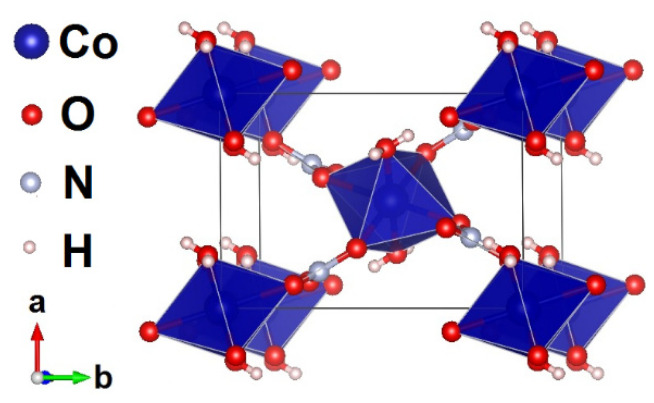
The crystal structure of Co(NO_3_)_2_⋅2H_2_O in polyhedral representation. The visualization of structure is provided through VESTA software [9].

The crystal structure of Co(NO_3_)_2_⋅2H_2_O is organized by almost regular square layers of Co^2+^ (3*d*^7^) ions, which are parallel to the (10$\bar{1}$) plane and connected via nitrate groups along the directions [111] and [1$\bar{1}$1], as shown in Figure 1. Water molecules coordinating cobalt ions are oriented by hydrogen atoms towards the neighboring planes.

Thermodynamic properties, i.e., magnetization, *M*, and specific heat, *C_p_*, of Co(NO_3_)_2_⋅2H_2_O have been measured on the collection of non-oriented microcrystals using various options of the “Quantum Design” Physical Properties Measurements System PPMS-9T (San Diego, CA, USA) in the temperature range 2–300 K in a magnetic field up to 9 T.

**Figure 2:**
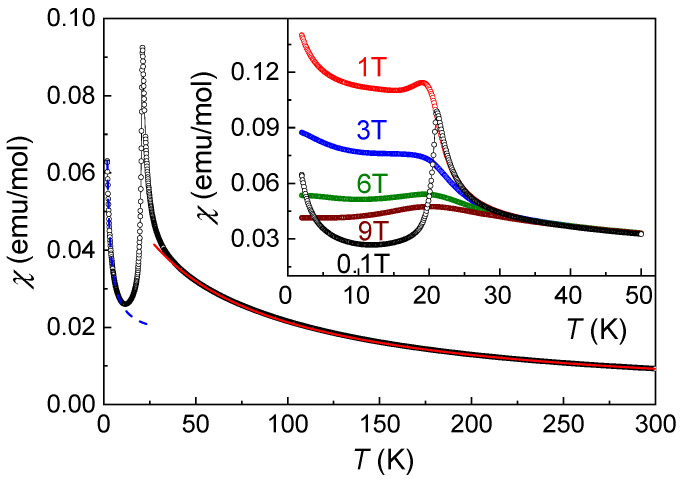
Temperature dependence of magnetic susceptibility in Co(NO_3_)_2_⋅2H_2_O taken at *µ*_0_*H* = 0.1 T. Solid line represents the Curie–Weiss fit. The dashed line represents the Curie fit of the low-temperature tail. Inset: *χ*(*T*) curves taken at various magnetic fields.

Temperature dependence of magnetic susceptibility, *χ* = *M*/*H*, of Co(NO_3_)_2_⋅2H_2_O taken in the field-cooled regime at *µ*_0_*H* = 0.1 T is shown in Figure 2. At elevated temperatures, this curve is evidence of the Curie–Weiss behavior

(1) $$\chi =\chi_{0}+\frac{C}{T-\Theta }$$

with temperature-independent term *χ*_0_ = 5.57⋅10^−4^ emu/mol, Curie constant *C* = 3.43 emu K/mol, and Weiss temperature *Θ* = − 57.5 K, as obtained from the fit in the range 200–300 K.

The value of *C* allows us to estimate the effective moment *µ_eff_* of Co^2+^ ions as

(2) $$\mu_{eff}={\left(8C\right)}^{1/2}$$

which equals 5.24 *µ_B_*.

Using the following equation:

(3) $$\mu_{eff}=g{\left[S\left(S+1\right)\right]}^{1/2}\mu_{B}$$

where *g*-factor is estimated as *g* = 2.69, which is reasonable for the high-spin state, *S* = 3/2, of Co^2+^ ions [10,11].

The negative value of *Θ* points to the predominance of antiferromagnetic exchange interactions. When approaching ordering temperature, magnetic susceptibility sharply increases, indicating strong pre-transitional fluctuations, and reaches a sharp peak at the Neel temperature, *T_N_* = 20.5 K. In variance with Heisenberg antiferromagnets, the magnetic susceptibility, *χ*, in Co(NO_3_)_2_⋅2H_2_O drops several times in magnitude below *T_N_*, which is the signature of Ising antiferromagnets with appreciable single-ion anisotropy [9]. The upturn of *χ* at the lowest temperatures can be attributed to defects/impurities not detected by X-ray diffraction. Their concentration is estimated at 2.5%. The origin of these defects is the extreme hygroscopicity of Co(NO_3_)_2_. Even short exposure to atmospheric moisture, inevitable for the mounting of the sample, results in the formation of metastable polyhydratesCo(NO_3_)_2_·*n*H_2_O, where *n* = 1, 2, 3, 4, 5, 6, 9. Thus, a direct comparison of the defect concentration found in X-ray diffraction and thermodynamics is questionable.

**Figure 3:**
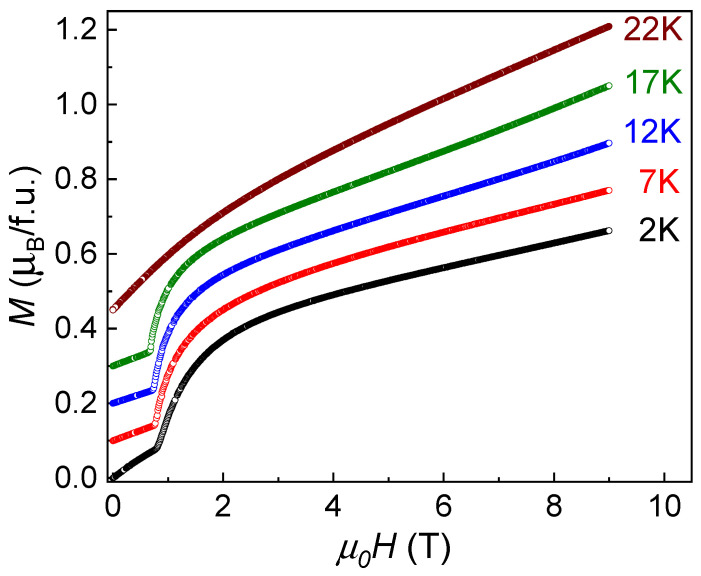
Field dependences of magnetization in Co(NO_3_)_2_⋅2H_2_O at various temperatures. The successive curves are shifted with respect to each other for clarity.

At *T* < *T_N_*, the field dependences of magnetization, *M*/*H*, present metamagnetic behavior inherent for Ising-type systems. Relevant curves taken at various temperatures are shown in Figure 3. At 2 K, the *M/H* curves shows a sharp transition at *µ*_0_*H_C_* = 0.76 T. No metamagnetism is seen at *T* > *T_N_*. Note that saturation magnetization, *M_sat_*, is expected at the level

(4) $$M_{sat}=ngS\mu_{B}$$

which equals ~ 4 *μ_B_* and can be reached in a very high magnetic field.

**Figure 4:**
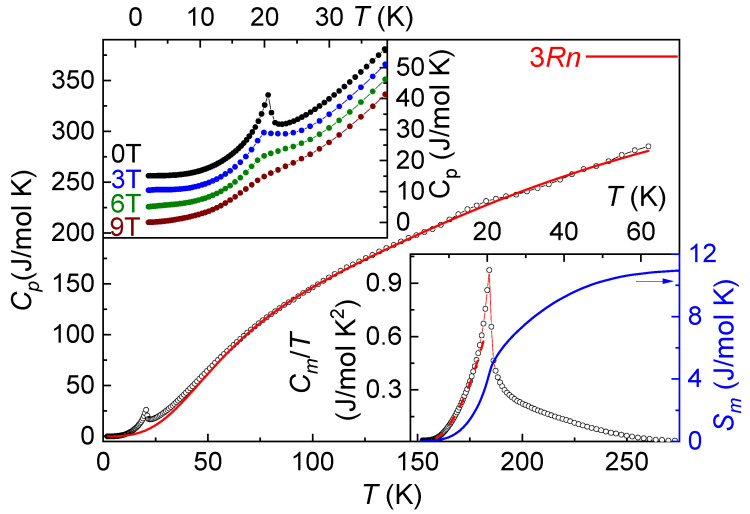
Temperature dependence of specific heat *C_p_* in Co(NO_3_)_2_⋅2H_2_O. The solid curve represents the lattice contribution. The horizontal line denotes the Dulong–Petit limit, 3*Rn*. The upper inset represents the *C_p_*(*T*) set obtained under various magnetic fields. The lower inset represents temperature dependences of both magnetic specific heat, *C_m_*, and magnetic entropy, *S_m_*. The dashed line is a fit of magnetic contribution.

The temperature dependency of specific heat, *C_p_*, in Co(NO_3_)_2_⋅2H_2_O is shown in Figure 4. It demonstrates the λ-type anomaly at the formation of long-range order at the Neel temperature, *T*_N_ = 20.5 K, which is smooth under the external magnetic field, as shown in the upper inset of Figure 4. The lattice contribution *C_lat_* has been calculated from the fit of high-temperature *C_p_* values by the sum of Debye [12] and Einstein [13] functions with corresponding weights *a_i_*, i.e., *Θ_D_* = 283 K (*a_D_* = 8.3) and *Θ_E_* = 813 K (*a_E_* = 7.3). The summation of these weights roughly equals 15, the number of atoms per formula unit. Magnetic entropy *S_m_* can be obtained by the integration of magnetic specific heat divided by temperature

(5) $$S_{m}=\int_{0}^{T}\frac{C_{m}}{T}dT$$

As shown in the lower inset to Figure 4, *S_m_* achieves ~ 11 J/mol K, which is close to the limit *S_m_* = *R*ln(2*S* + 1) = 11.5 J/mol, where *R* is the universal gas constant.

Magnetic specific heat of an antiferromagnet with the gap, *E_g_*, in magnon excitation spectrum is described by the following equation [13]:

(6) $$C_{m}=\alpha T^{3}exp\left(\frac{-E_{g}}{k_{B}T}\right)$$

The fit of *C_m_*(*T*)/T dependence shown by the dotted line in the lower inset to Figure 4 allows for an estimate of *E_g_* = 9.4 K and *α* = 2.6 × 10^−3^ J/molK^4^. Leading exchange interaction *J* relates to *α* [14]

(7) $$J=\left(R^{4/3}/2S\right)\times {\left(s_{af}/\alpha \right)}^{1/3}$$

where *s*_af_ is the coefficient calculated for *fcc* lattice as *s_af_* = 2.825 10^−2^ [13]. According to this estimation, *J* = 12.4 K.

## 3. First-Principles Calculations

In order to gain microscopic understanding of Co(NO_3_)_2_·2(H_2_O),we carried out first-principles density functional theory (DFT) [15] calculations using plane wave basis as implemented within Vienna Ab initio Simulation Package (VASP, 5.4.1; 2015; G. Kresse, J. Furthmuller; Wien; Austria) [16]. For the self-consistent field calculation in the plane-wave basis, energy cutoffs of 500 eV and 4 × 4 × 4 Monkhorst–Pack k-points mesh for Co(NO_3_)_2_·2(H_2_O) compound were found to provide a good convergence of the total energy (*E* = 10^−5^ eV). The exchange-correlation functional for the self-consistent calculations was chosen as that of generalized gradient approximation (GGA) implemented following the Perdew–Burke–Ernzerhof prescription [17]. To check the missing correlation energy at Co sites beyond GGA, calculations with supplemented Hubbard U (GGA + U) were carried out [18].

**Figure 5:**
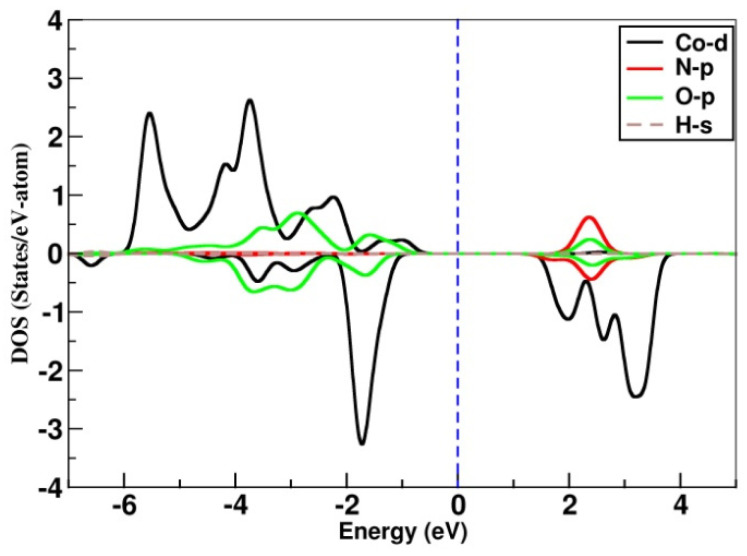
Spin-polarized density of state (DOS) projected onto Co-d, O-p, N-p and H-s states. Zero energy corresponds to Fermi energy in generalized gradient approximation. Positive and negative values correspond to the states in majority and minority spin channels.

The spin-polarized density of states, obtained in a self-consistent spin-polarized GGA + U calculation, projected onto Co-*d*, N-*p*, O-*p*, and H-*s* states, is shown in Figure 5. We also find that the octahedral crystal field split Co-*t*_2g_ and -*e*_g_ states are completely filled in the majority spin-channel and partially filled in the minority spin-channel, suggesting the stabilization of high-spin nominal Co^+2^ or *d*^7^ valence of Co. The O-*p* state is found to be mostly occupied, which explains the nominal O^−2^ valence state. The O-*p* state shows finite, non-zero hybridization with Co-*d* state close to Fermi energy, which contributes to the super-exchange path of magnetic interaction. The calculated magnetic moments at Co and O sites are found to be 2.72 *µ_B_* and 0.025 *µ_B_*_,_ with the rest of the moment sitting at neighboring N-and H-sites with a total magnetic moment of 3 *μ_B_* per formula unit. A rather large magnetic moment was observed at the O site, indicating strong Co-O covalency which contributes to the super-exchange path connecting two Co sites.

## 4. Magnetic Interactions

**Figure 6:**
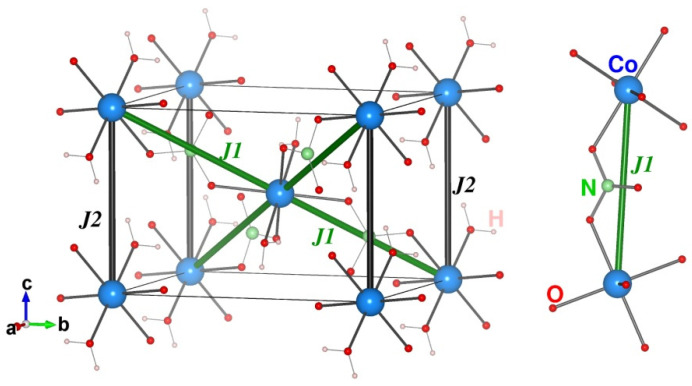
Exchange paths for dominant magnetic interactions *J1* (in green) and *J2* (in black) in Co(NO_3_)_2_·2(H_2_O).

In order to calculate the various Co-Co magnetic exchange interactions present in the compound, we carried out total energy calculation for different spin arrangements in GGA + U scheme and found the dominant magnetic exchanges [18]. For this purpose, we made a supercell of dimension 1 × 1 × 2 and Monkhorst–Pack k-points mesh 4 × 4 × 2 of the compound, giving rise to 4 Co atoms in the unit cell. The solutions of the calculated GGA + U energies of different spin arrangements for the considered configurations and the energies gave the estimation of *J*’s. Following the estimates of constrained DFT calculations [18], we have set the U values at Co sites to be 4 eV. The paths for dominant magnetic interactions for Co(NO_3_)_2_·2(H_2_O) are shown in Figure 6. We see that in Co(NO_3_)_2_·2(H_2_O) the intra-layer interaction *J1* is mediated by Co d-O p-N p-O p-Co d super-exchange path while, there is no direct connection for inter-layer interaction *J2*. The dominant effective Co-Co hopping antiferromagnetic interactions are *J1* = 9.86 K and *J2* = 0.46 K. In our calculation, the Co^2+^ ion has an easy plane anisotropy with the plane perpendicular to the three-fold rotational axis *x*′. Therefore, the single-ion anisotropy term can be expressed as: H_SIA_ = *DS*^2^*x*′. To evaluate *D*, we considered the four spin states in which the spin directions for site Co1 are along *x*′, −*x*′, *y*′ and −*y*′, with the spins at the Co2 site along the *z*′ direction. Total energy calculations were carried out for the four spin directions within the GGA + U + Spin Orbit Coupling (SOC) scheme of calculations, resulting in energies *E1*, *E2*, *E3* and *E4*. From these calculations *D* is obtained as, *D* = (*E1* + *E2* − *E3* – *E4*)/*2S*^2^. Total energy calculations were performed including SOC effects because the single-ion anisotropy is a consequence of SOC. The single-ion anisotropy *D*, as estimated by total energy calculation [19], is 1K.

**Figure 7:**
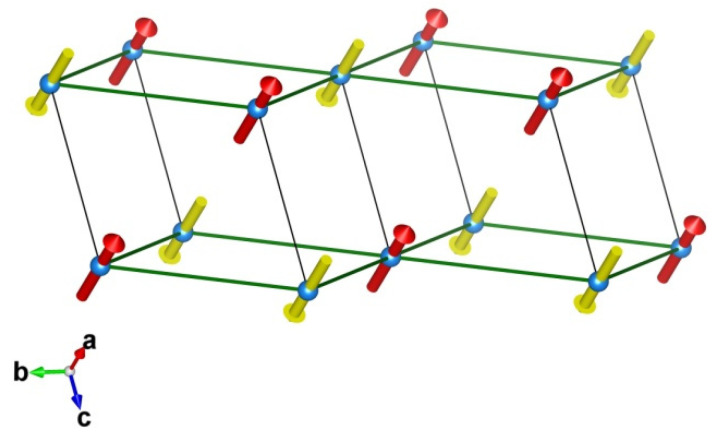
The magnetic model of Co(NO_3_)_2_·2(H_2_O).

Based on the nature of the magnetic exchange interactions present in the compound, the underlying magnetic model is shown in Figure 7. As found, the magnetic model connects the four nearest neighbor in-plane magnetic Co^2+^ ions antiferromagnetically and the two out-of-plane Co^2+^ ions also antiferromagnetically, giving rise to a three-dimensionally connected antiferromagnetic structure. The underlying magnetic model is a magnetically non-frustrated spin model. A collinear antiferromagnetically ordered ground state is thus expected.

Focusing on the calculated magnetic interactions, we found that the dominant intra-layer interaction is *J1* while the inter-layer interaction *J2* is twenty times smaller. Therefore, *J1* and *J2* form a weakly antiferromagnetically coupled layered structure. We note that estimated tiny values of *J2* and *D* are close to the accuracy limit of DFT, and the precise numerical value may not be trustworthy.

Calculated values of exchange interaction parameters allow for estimating the Weiss temperature in Co(NO_3_)_2_⋅2H_2_O using the mean field expression:

(8) $$\Theta =\frac{S\left(S+1\right)}{3}\underset{i}{\sum }z_{i}J_{i}$$

where *z* is the number of nearest neighbors equal to 4 for *J1* and 2 for *J2*. The calculated value of *Θ^calc^* = −50.5 K is in good correspondence with the experimentally found value *Θ* = −57.5 K.

Experimentally, we found that the value of metamagnetic phase transition, *µ_0_H_C_* = 0.76 T, can be put into correspondence with the calculated value of the single-ion anisotropy [20]:

*gSµ_B_µ*_0_*H_C_* = *2D* (9)

At D = 1 K, the calculated field of metamagnetic transition isµ_0_H_C_^calc^ = 0.74 T.

The mean field theory largely overestimated the Neel temperature in Co(NO_3_)_2_⋅2H_2_O. However, strong spin–orbital coupling in Co^2+^ ions allows us to consider their effective moment, M_eff_ = 1/2. In this case, the Neel temperature estimated through the formula was appropriate for the layered magnetic systems [21].

(10) $$T_{N}=\frac{4\pi J_{1}M_{eff}^{2}}{\ln(J_{1}\pi^{2}/J_{2})}$$

results in *T_N_^calc^* = 23.2 K, which is in good correspondence with experimentally found value *T_N_* = 20.5 K.

Overall, the magnetic behavior of Co(NO_3_)_2_⋅2H_2_O is influenced by the single-ion anisotropy of Co^2+^ ions. Besides the pronounced drop of magnetic susceptibility at *T* < *T_N_*, it defines the field of metamagnetic transition. At this field, the magnetic moments of Co^2+^ ions initially oriented antiparallel to the external magnetic field flip to the field direction.

## 5. Conclusions

InCo(NO_3_)_2_⋅2H_2_O, we find an example of the system evidencing Ising-like behavior. Two water molecules introduced into the crystal structure of Co(NO_3_)_2_ play the role of chemical scissors, cutting the original framework structure into layers coupled through hydrogen bonds. Properties of Co(NO_3_)_2_⋅2H_2_O are in sharp contrast with those observed in waterless Co(NO_3_)_2_ [22]. The behavior of Co(NO_3_)_2_ is of the Heisenberg type, where the frustration of exchange interactions is lifted by the formation of a 120° noncollinear structure. Usually, this is not the case for Ising antiferromagnets. In Co(NO_3_)_2_, however, the 120° motif appears due to the mutual orientation of local easy axes in CoO_6_ octahedra. While the magnetic structures of both Co(NO_3_)_2_ and Co(NO_3_)_2_⋅2H_2_O are not established yet experimentally, we believe that the simple collinear antiferromagnetic arrangement is realized in Co(NO_3_)_2_⋅2H_2_O contrary to the exotic chiral noncoplanar ferrimagnet of the inverted umbrella type in Co(NO_3_)_2_.

## Data Availability

Data available on Request.

## Supplementary Materials

The following supporting information can be downloaded at: https://www.mdpi.com/article/10.3390/ma15207066/s1, Figure S1: X-ray pattern of polycrystalline Co(NO_3_)·2(H_2_O)_2_ sample. High background level associated with the use of X-ray amorphous adhesive tape. In addition, XRD Co-containing samples with using Cu (Kα) radiation gives a large background due to secondary radiation darkening the diffraction pattern.

## References

[1] Stryjewski E., Giordano N. (1970). Metamagnets. Adv. Phys..

[2] Onsager L. (1944). Crystal Statistics. I. A two-dimensional model with an order-disorder transition. Phys. Rev..

[3] Mermin N.D., Wagner H. (1966). Absence of ferromagnetism or antiferromagnetism in one- or two-dimensional isotropic Heisenberg models. Phys. Rev. Lett..

[4] Gong C., Li L., Li Z., Ji H., Stern A., Xia Y., Cao T., Bao W., Wang C., Wang Y. (2017). Discovery of intrinsic ferromagnetism in two-dimensional van der Waals crystals. Nature.

[5] Cortie D.L., Causer G.L., Rule K.C., Fritzsche H., Kreuzpaintner W., Klose F. (2019). Two-dimensional magnets: Forgotten history and recent progress towards spintronic applications. Anv. Funct. Mater..

[6] Wildner M., Giester G., Lengauer C.L., Zemann J. (2012). Investigation of low-hydrated metal (II) nitrates. Synthesis and crystal structures of Zn(NO_3_)_2_⋅2H_2_O and M(NO_3_)_2_⋅2H_2_O (M = Mg, Mn, Co, Ni). Z. Kristallogr..

[7] Berger L., Friedberg S.A. (1964). Low-Temperature Magnetic Susceptibilities of the Hydrated Nickel Nitrates. Phys. Rev. B.

[8] Schmidt V.A., Friedberg S.A. (1970). Metamagnetism of Ni(NO_3_)_2_⋅2H_2_O. Phys. Rev. B.

[9] Momma K., Izumi F. (2011). VESTA 3 for three-dimensional visualization of crystal, volumetric and morphology data. J. Appl. Cryst..

[10] Osaki K., Uryu N. (1975). Anisotropic g-factors of Co^2+^ ions in complex salts. J. Phys. Soc. Jpn..

[11] Foglio M.E., Barberis G.E. (2006). Study of Co^2+^ in different crystal field environments. Bras. J. Phys..

[12] Goetsch R.J., Anand V.K., Pandey A., Johnston D.C. (2012). Structural, thermal, magnetic, and electronic transport properties of the LaNi_2_(Ge_1−x_P_x_)_2_ systemPhys. Phys. Rev. B.

[13] Tari A. (2003). The Specific Heat of Matter at Low Temperatures.

[14] Lashley J.C., Stevens R., Crawford M.K., Boerio-Goates J., Woodfield B.F., Qiu Y., Lynn J.W., Goddard P.A., Fisher R.A. (2008). Specific heat and magnetic susceptibility of the spinels GeNi_2_O_4_ and GeCo_2_O_4_. Phys. Rev. B.

[15] Kohn W., Sham L.J. (1965). Self-Consistent Equations Including Exchange and Correlation Effects. Phys. Rev. A.

[16] Kresse G., Furthmuller J. (1996). Efficient iterative schemes for ab initio total-energy calculations using a plane-wave basis set. Phys. Rev. B.

[17] Perdew J.P., Burke K., Ernzerhof M. (1996). Generalized Gradient Approximation Made Simple. Phys. Rev. Lett..

[18] Anisimov V.I., Solovyev I.V., Korotin M.A., Czyzyk M.T., Sawatzky G.A. (1993). Density-functional theory and NiO photoemission spectra. Phys. Rev. B.

[19] Xiang H.J., Kan E.J., Wei S.H., Whangbo M.H., Gong X.G. (2011). Predicting the spin-lattice order of frustrated systems from first principles. Phys. Rev. B.

[20] Kalita V.M., Loktev V.M. (2005). On the sequence of quantum (meta)magnetic transitions in Ising antiferromagnets with single-ion anisotropy. Low. Temp. Phys..

[21] Irkhin V.Y., Katanin A.A. (1997). Critical behavior and the Néel temperature of quantum quasi-two-dimensional Heisenberg antiferromagnets. Phys. Rev. B.

[22] Danilovich I.L., Deeva E.B., Bukhteev K.Y., Vorobyova A.A., Morozov I.V., Volkova O.S., Zvereva E.A., Maximova O.V., Solovyev I.V., Nikolaev S.A. (2020). Co(NO3)2as an inverted umbrella-type chiral noncoplanar ferrimagnet. Phys. Rev. B.

