# Supplementary material for: Ising-like Magnetism in Quasi-Two-Dimensional Co(NO3)2·2H2O
Source: Materials (Basel). 2022 Oct 11;15(20):7066. doi: 10.3390/ma15207066 (PMC9605630; doi:10.3390/ma15207066)
Supplement: Supplementary file 1 [file materials-15-07066-s001.zip › materials-1934801-supplementary.pdf]

Supplementary Materials

Ising-Like Magnetism in Quasi-Two-Dimensional $Co(NO_3)_2·2H_2O$

Anna A. Vorobyova [1,2,*], Igor L. Danilovich [1], Igor V. Morozov [1,2], Alexander N. Vasiliev [1,2], Olga S. Volkova [1,2,*], Asif Iqbal [3], Badiur Rahaman [3] and Tanusri Saha-Dasgupta [4]

1 Department of Low Temperature Physics and Superconductivity, M.V. Lomonosov Moscow State University, Moscow 119991, Russia
2 Quantum Functional Materials Laboratory, National University of Science and Technology "MISiS", Moscow 119049, Russia
3 Department of Physics, Aliah University, Kolkata 700156, India
4 Department of Condensed Matter, S.N. Bose National Centre for Basic Sciences, Kolkata 700106, India
* Correspondence: vorobyova.anna9@gmail.com (A.A.V.); os.volkova@yahoo.com (O.S.V.)

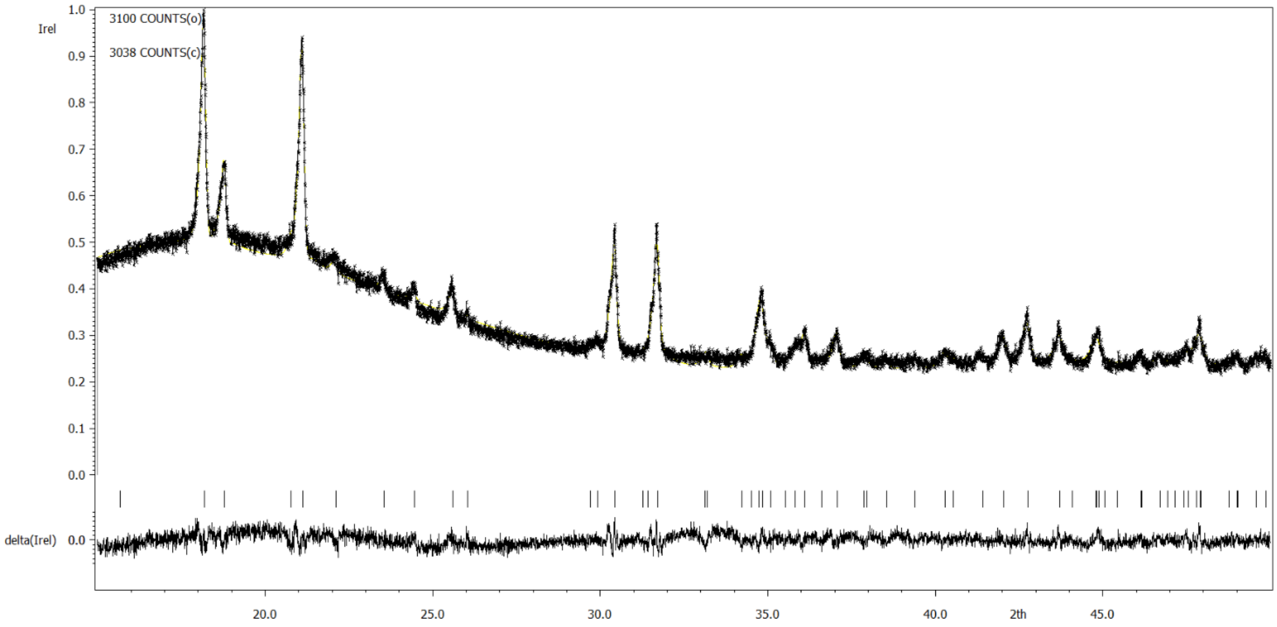

Figure S1. X-ray pattern of polycrystalline $Co(NO_3)_2(H_2O)_2$ sample. High background level associated with the use of X-ray amorphous adhesive tape. In addition, XRD Co-containing samples with using Cu (K_α) radiation gives a large background due to secondary radiation darkening the diffraction pattern.
